# A Practical Approach to the Percutaneous Treatment of Iatrogenic Aorto-coronary Dissection

**DOI:** 10.2174/1874192401812010050

**Published:** 2018-04-25

**Authors:** George Kassimis, Tushar Raina

**Affiliations:** Department of Cardiology, Cheltenham General Hospital, Gloucestershire Hospitals NHS Foundation Trust, Gloucester, United Kingdom

**Keywords:** Iatrogenic, Aortic dissection, Cardiac catheterization, Percutaneous coronary intervention, Acute coronary syndromes, Guiding catheter

## Abstract

Catheter-induced aortic dissection during coronary angiography and Percutaneous Coronary Intervention (PCI) is a relatively infrequent, but potentially life-threatening complication. Patients who suffer this complication may require emergency aortic surgery. More recently, reports of treating the ostium of the dissected coronary artery have emerged as an alternative therapeutic option. In this article we describe two cases of extensive guide catheter induced dissection and their successful treatment using PCI and provide a concise overview of the available literature.

## 1 INTRODUCTION

Aortic dissection during Percutaneous Coronary Intervention (PCI) is a rare, but potentially life-threatening complication which requires early recognition and emergency treatment. The treatment may involve conservative management, surgical repair or even bailout PCI. The successful percutaneous treatment of this complication during PCI for stable angina has been previously reported [1]. In this series, we describe two guide catheter induced dissections of the Right Coronary Artery (RCA) during PCI for acute coronary syndromes. In the first case, the dissection involved the aortic root and in the second case, there was retrograde extension to the RCA ostium sparing the ascending aorta. We describe the successful percutaneous treatment in both cases and review the available literature.

### 1.1 Case 1

**Fig. (1):**
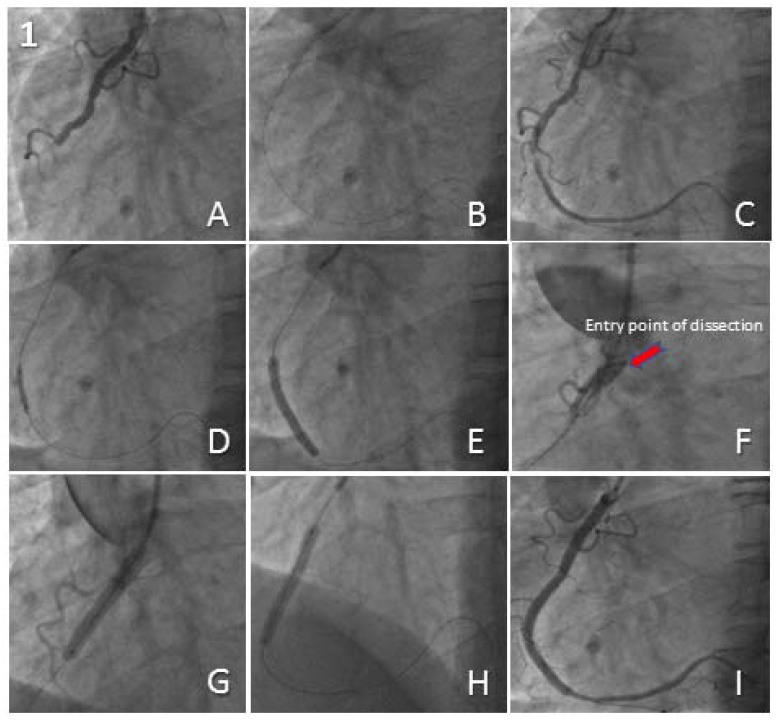
Extensive right coronary artery and ascending aortic root dissection successfully treated through proximal to distal stenting from the ostium.

A 52-year-old male with a previous history of hypertension was admitted to our centre with an inferior ST-segment Elevation Myocardial Infarction (STEMI). Transradial Coronary Angiography (TCA) demonstrated a thrombotic occlusion in the mid portion of a dominant RCA with TIMI 0 flow (Fig. **1A**). Primary PCI was carried out using a 6F JR4 Guiding Catheter (GC) and a ChoICE^TM^ PT (Boston Scientific, Boston, MA, USA) guide wire. Thrombus aspiration was performed using 6F Export^®^AP (Medtronic Inc, Minneapolis, Minnesota, USA) aspiration catheter which restored TIMI 3 flow (Fig. **1B**, **C**). Pre-dilatation was performed using a 2.5mm x 15mm semi-compliant balloon (Fig. **1D**). A Zotarolimus-eluting Stent (ZES) measuring 3.5mm x 34mm was implanted to treat the diseased segment (Fig. **1E**). Unfortunately the inadvertent deep engagement of the GC during retrieval of the deflated stent balloon resulted in an extensive RCA and ascending aortic dissection (Fig. **1F**).

This was successfully sealed by the implantation of three more ZES measuring 4.0mm x 12mm, 4.0mm x 30mm and finally 3.5mm x 38mm from proximal to distal (Fig. **1G**, **H**) with limited extravasation of radiographic contrast into the ascending aorta (Fig. **1I**). Sealing of the aortic dissection and successful salvage of the RCA was possible due to prompt recognition and stenting of the RCA ostium. Subsequent CT aortography revealed a localised collection of radiographic contrast in the ascending aorta sparing the arch and the aortic valve. Transthoracic Echocardiography (TTE) did not reveal any evidence of pericardial collection and no evidence of aortic regurgitation. His remaining hospital stay was uneventful and he was discharged home 3 days later.

### 1.2 Case 2

**Fig. (2):**
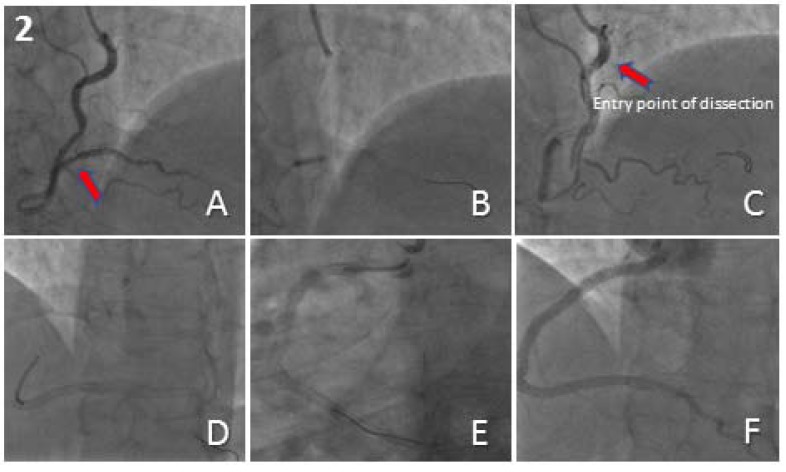
Spiral dissection of the right coronary artery successfully treated through distal to proximal stenting up to the ostium.

A 55-year-old female with a previous history of hypertension was admitted to our centre with a non ST-segment elevation MI. TCA revealed a severe focal distal stenosis of a dominant RCA (Fig. **2A**). A Fractional Flow Reserve (FFR)-guided PCI to the distal RCA was performed using a 6F JR4 GC. Pre-dilatation was performed using a 2.0mm x 12mm semi-compliant balloon. An Everolimus-eluting Stent (EES) measuring 2.5mm x 16mm was implanted to treat the disease segment (Fig. **2B**). Stent delivery proved difficult and required deep engagement of the GC into a tortuous proximal RCA, which unfortunately resulted in an extensive propagating dissection of the RCA involving the ostium but sparing the aortic root, with transient loss of antegrade flow (Fig. **2C**). The dissection was successfully treated through PCI under fluoroscopic guidance without the use of any radiographic contrast. Four EES measuring 4.0mm x 32mm, 4.0mm x 32mm, 4.0mm x 28mm and 4.5mm x 20mm were implanted from distal to proximal (Fig. **2D**, **2E**) with an excellent final angiographic result and TIMI 3 flow (Fig. **2F**). CT aortography confirmed the absence of aortic root involvement and TTE demonstrated normal cardiac structure and function. Her subsequent hospital stay was uneventful and she was discharged home 2 days later.

## 2 RESULTS & DISCUSSION

Iatrogenic aorto-coronary dissection is rare with a reported incidence of approximately 0.062% of all cardiac catheterization procedures. The incidence of those involving the coronary tree alone has been estimated at 0.039% (0.006% during coronary angiography and 0.098% during interventional procedures) [2, 3].

Although the exact mechanism for the occurrence and propagation of iatrogenic aorto-coronary dissection remains unclear, it is believed that the entry point originates within the coronary dissection with retrograde extension of the subintimal space into the aortic root. This can occur due to trauma caused by the tip of the diagnostic or guiding catheter, subintimal entry of a stiff guidewire or due to balloon dilatation.

The risk factors for aorto-coronary dissection include age, diabetes mellitus, hypertension, previous coronary artery bypass grafting, concomitant acute myocardial infarction, atherosclerotic burden and any underlying structural weakness of the tunica media [2-5]. Both patients described above although young, were suffering an acute myocardial infarction and had a previous history of hypertension. Neither had any evidence of aortopathy or predisposition to vascular injury due to Marfan’s syndrome or other causes of medial necrosis.

Aorto-coronary dissections involve the RCA more than the Left Main Coronary Artery (LMCA) [3]. This predisposition maybe due to the differences in the histological structure of the ostia of the RCA and the LMCA [2, 6]. The dissection usually occurs during the use of GCs and 6F diagnostic catheters. All access sites and catheter curves have been shown to be related with this complication. The most frequent shape of catheter responsible is Amplatz, followed by Judkins through the transradial approach and Judkins followed by Amplatz through the transfemoral approach [3].

Dunning *et al*. proposed a classification of iatrogenic aortic dissection into in 3 grades: type 1, dissection limited to the sinuses of Valsalva; type 2, dissection of the ascending aorta beyond the sinuses but < 4 cm; and type 3, dissection ≥ 4 cm [5].

When a coronary artery is involved as the entry point, it can usually be successfully sealed using conventional stents [3, 7], or covered stents if there is associated extravasation into the pericardial space [8]. The use of intravascular ultrasound and/or fluoroscopy guidance during PCI is advisable, as repeated radiographic contrast injection may result in extension of the dissection flap [9]. Progression of the aortic dissection with haemodynamic instability, acute aortic regurgitation, haemopericardium or intractable chest pain are indications for urgent surgical intervention [2]. The integrity of the aortic valve should be assessed through echocardiography in all cases. Conservative management is a reasonable option for haemodynamically stable localized aortic dissections which should be monitored using serial CT aortic imaging and TTE [10, 11].

## CONCLUSION

Percutaneous intervention of the RCA ostium is an acceptable alternative to surgical repair for the treatment of limited iatrogenic aorto-coronary dissection. If the aortic root is involved, the aim should be to seal the entry site of the dissection with PCI first, to prevent any further aortic extension. If the aortic root is spared, PCI using distal to proximal stenting under fluoroscopic guidance, without the use of radiographic contrast, can be safely performed with excellent results.
